# PERK-Mediated Unfolded Protein Response Signaling Restricts Replication of the Tick-Borne Flavivirus Langat Virus

**DOI:** 10.3390/v12030328

**Published:** 2020-03-18

**Authors:** Tyler G. Lewy, Danielle K. Offerdahl, Jeffrey M. Grabowski, Eliza Kellman, Luwanika Mlera, Abhilash Chiramel, Marshall E. Bloom

**Affiliations:** 1 Biology of Vector-Borne Viruses Section, Laboratory of Virology, Rocky Mountain Laboratories, NIAID/NIH, 903 S. 4th St, Hamilton, MT 59840, USA; tlewy@rockefeller.edu (T.G.L.); offerdahld@niaid.nih.gov (D.K.O.); ekellman@usc.edu (E.K.); luwanikamlera@email.arizona.edu (L.M.); 2 Innate Immunity and Pathogenesis Section, Laboratory of Virology, Rocky Mountain Laboratories, NIAID/NIH, 903 S. 4th St, Hamilton, MT 59840, USA; abhilash.chiramel@nih.gov

**Keywords:** tick-borne flavivirus, unfolded protein response, PERK, Langat virus, autophagy

## Abstract

The unfolded protein response (UPR) maintains protein-folding homeostasis in the endoplasmic reticulum (ER) and has been implicated as both beneficial and detrimental to flavivirus infection. Protein kinase R (PKR)-like endoplasmic reticulum kinase (PERK), a sensor of the UPR, is commonly associated with antiviral effects during mosquito-borne flavivirus (MBFV) infection, but its relation to tick-borne flavivirus (TBFV) infection remains largely unexplored. In this study, we identified changes in UPR and autophagic activity during Langat virus (LGTV) infection. LGTV robustly activated UPR and altered autophagic flux. Knockdown of endogenous PERK in human cells resulted in increased LGTV replication, but not that of closely related Powassan virus (POWV). Finally, on examining changes in protein levels of components associated with UPR and autophagy in the absence of PERK, we could show that LGTV-infected cells induced UPR but did not lead to expression of C/EBP homologous protein (CHOP), an important downstream transcription factor of multiple stress pathways. From these data, we hypothesize that LGTV can antagonize other kinases that target eukaryotic initiation factor 2α (eIF2α), but not PERK, implicating PERK as a potential mediator of intrinsic immunity. This effect was not apparent for POWV, a more pathogenic TBFV, suggesting it may be better equipped to mitigate the antiviral effects of PERK.

## 1. Introduction

Flaviviruses are a diverse family of small, enveloped viruses, transmitted primarily by arthropod vector hosts. These agents require several cellular systems and pathways to complete their lifecycle [1]. The majority of studies into flavivirus–host cell interactions have focused on the mosquito-borne flaviviruses (MBFVs), such as West Nile virus (WNV) and dengue virus (DENV). However, the incidence of tick-borne flaviviruses (TBFVs) such as Powassan virus (POWV) [2,3,4,5] and tick-borne encephalitis virus (TBEV) [6,7] is rising due to a number of ecological and climatic factors [8]. POWV and TBEV can cause debilitating encephalitic disease resulting in death or long-term neurological sequelae and have no specific treatments beyond palliative care [9]. The related and naturally attenuated TBFV Langat virus (LGTV), has proven a valuable model system to study the highly neurovirulent TBFVs such as POWV and TBEV [10].

Flavivirus genome replication and translation occurs primarily on membranes of the endoplasmic reticulum. This process places significant stress on the infected cell, because translation of the viral polyprotein is not subject to the same stringent intrinsic control as are most host proteins. Cells respond to the accumulation of proteins in the endoplasmic reticulum (ER) by activation of the unfolded protein response (UPR). The UPR is an ancient system used by eukaryotic cells to respond to translational stress, and orthologous systems are present in most eukaryotes, spanning yeast to humans [11,12,13]. The UPR maintains conditions within the ER lumen that are ideal for appropriate protein folding using a luminal chaperone, binding immunoglobulin protein (BiP) and three proteins embedded in the ER membrane, including PKR-like ER Kinase (PERK) [14]. In non-stress situations, BiP non-covalently associates with these proteins keeping them in a dormant state. However, in the presence of unfolded peptides, BiP dissociates from these sensors and permits downstream signaling [15]. PERK phosphorylates eukaryotic translation initiation factor 2α (eIF2α), which leads to rapid attenuation of global protein synthesis. eIF2α phosphorylation is common to multiple cellular stress pathways including that of protein kinase R (PKR), a sensor of dsRNA commonly activated during viral infection. Some transcripts are able to bypass the eIF2α-mediated translational block, including activating transcription factor 4 (ATF4), which subsequently activates expression of CCAAT-enhancer-binding protein homologous protein (CHOP), growth arrest and DNA damage inducible protein 34 (GADD34), and other proteins responsible for modulating amino acid metabolism, redox homeostasis, and activation of apoptosis [16,17]. PERK, along with the other two sensors, coordinately functions to maintain ER homeostasis.

Several families of enveloped viruses, including flaviviruses, activate the UPR [18,19,20,21,22]. MBFVs, including DENV, WNV, and Japanese encephalitis virus (JEV), increase expression of BiP, a common marker of UPR activity. It is undetermined whether this is due to some direct action by the virus itself or is simply a response to the high peptide burden of infection [23,24,25]. PERK knockout or knockdown studies using mouse embryonic fibroblast (MEF) cells have shown that PERK plays a largely antiviral role in MBFV replication [25,26,27]. One study, however, suggested that PERK might actually aid DENV replication by promoting autophagosome formation and turnover [28]. The TBFVs have been the subject of less inquiry. TBEV has been shown to activate the inositol requiring enzyme 1 (IRE1) arm of the UPR leading to priming of the innate immune system, but no role has yet been assigned to PERK [29,30].

The UPR is not the only homeostatic system the cell employs to regulate protein folding. Autophagy is another highly conserved cellular pathway used to manage cell resources via the recycling of intracellular components, ranging from cytosolic peptides to entire organelles. Three types of autophagy are known to occur in mammalian cells: microautophagy, macroautophagy, and chaperone-mediated autophagy (CMA). While microautophagy and CMA degrade substrates on a peptide level, macroautophagy is capable of recycling large amounts of cellular material, including damaged organelles and membranes. When macroautophagy occurs, the cell assembles a double-membraned structure called an autophagosome. As this structure forms, the cytosolic protein, microtubule-associated protein light chain 3 (LC3), is lipidated with phosphatidylethanolamine and used to decorate the interior of the forming autophagosome where it serves as an attachment site for cargo receptors, such as SQSTM1/p62 [31,32]. LC3 is a commonly assayed marker of autophagic activity. Because only the lipidated form (LC3-II) can be inserted into autophagosome membranes, an autophagic state can be inferred by comparing the inactive unlipidated (LC3-I) and active lipidated (LC3-II) forms [33]. Once the autophagosome has engulfed the target and closed, it fuses with a lysosome to create the autolysosome, in which the contents are degraded. Autophagy is crucial not only for component recycling in the cell but also for directly modulating innate immune function, including NF-κB response and major histocompatibility complex (MHC) 1 and 2 antigen presentation [34]. Autophagy has different effects on MBFVs [35] ranging from pro-viral (JEV, DENV, and Zika virus [36,37,38,39]), to antiviral (DENV [40]), to no effect (WNV [41,42]) depending on cell type and stage of infection.

In this paper, we explored the role of PERK in the TBFV replication cycle, using LGTV as a model. First, we measured UPR signaling and PERK-mediated activity by western blot. We next generated a PERK-knockdown HEK293T cell line using CRISPR technology and used it to interrogate the function of PERK in viral infection. Finally, we looked at several upstream and downstream targets of PERK as well as the autophagy pathway to determine potential mechanisms of restriction. We additionally found that LGTV is able to antagonize CHOP expression independent of PERK, providing preliminary evidence of PERK as an intrinsic immune factor against TBFV infection.

## 2. Materials and Methods

### 2.1. Cell Culture and Viruses

HEK293T and Vero cells (ATCC, Manassas, VA, USA) were maintained in Dulbecco’s minimal essential media ((DMEM), Life Technologies, Carlsbad, CA, USA) supplemented with 50 μg/mL gentamycin and 10% fetal bovine serum (FBS) at 37 °C in 5% CO_2_.

Langat virus (TP21 strain) and Powassan virus (lineage I LB Prototype strain; originally obtained from Robert Tesh, University of Texas Medical Branch) were prepared as previously described [43,44].

### 2.2. Antibodies

Antibodies used in this study are as follows: PERK (Cell Signaling, C33E10, Danvers, MA, USA), BiP (Cell Signaling, C50B12, Danvers, MA, USA), CHOP (Cell Signaling, L63F7, Danvers, MA, USA), β-actin (Sigma Aldrich, A5441, St. Louis, MO, USA), LC3 (Enzo Life Sciences, 5F10, Farmingdale, NY, USA), LGTV envelope (E) (a kind gift from Dr. Connie Schmaljohn, USAMRID, Fort Detrick, Frederick, MD, USA, 11H12), LGTV nonstructural protein 3 (NS3) (custom polyclonal prepared by Aves Labs (Tigard, OR, USA), sequence: CZRDIREFVSYASGRR), ATF6 (Abcam, ab122897, Cambridge, MA, USA), Alexa Fluor 594-conjugated anti-mouse and Alexa Fluor 647-conjugated anti-chicken secondaries (Thermo Fisher, Waltham, MA, USA), HRP-conjugated anti-mouse and anti-rabbit secondaries (Jackson ImmunoResearch, West Grove, PA, USA).

### 2.3. Generation of CRISPR Knockdown Cell Line

HEK293T cells were seeded in six-well plates to a density of 4 × 10^5^ cells per well. A plasmid containing the *Cas9* gene, mDASHER-GFP, and sgRNA targeting exon 5 of PERK (Hs:2: 88,890,383–88,890,422) (Atum, Newark, CA, USA) was transfected at 1 µg per well with Effectene transfection reagent (Qiagen, Germantown, MD, USA) according to the manufacturer’s protocols. The cells were incubated in the transfection mixture at 37 °C for 24 h. Transfected cells were examined for GFP expression using an Axio Vert.A1 microscope (Zeiss, White Plains, NY, USA) equipped with a PhotoFluor LM-75 light source (89 North, Williston, VT, USA) and an ET-GFP (FITC/Cy2) filter (Chroma, Bellows Falls, VT, USA). The transfection mixture was removed and replaced with fresh media. Following another 24 h incubation, transfected cells were pooled and plated into single-cell colonies on 96-well plates. Following eight days of growth, isolated colonies were selected and expanded. Subclones were evaluated for PERK expression using western blot.

Additionally, the nucleotide sequence at the putative edit site in this cell line was characterized at both a low (p. 13) and high passage (p. 108) number to monitor the knockout. Briefly, genomic DNA was extracted (DNAzol, Thermo Scientific, Waltham, MA, USA) from a low or high passage number wild-type (WT) or CRISPR-treated cell and the editing locus was amplified by PCR (forward primer: 5′- GTGGAATTTCAGTGTTGGCCACTTTGAAC -3′, reverse primer: 5′- TGGTGTTAG GTACCTGGTACTCCC -3) producing an expected product of 248 bp (Phusion, New England Biolabs, Ipswich, MA, USA). The DNA was purified (QIAquick PCR purification kit, Qiagen, Germantown, MD, USA), quantified (Qubit dsDNA HS Assay, Thermo Fisher, Waltham, MA, USA), and sequenced using MiSeq technology at Massachusetts General Hospital, Center for Computational and Integrative Biology. These cells will be referred to as PERK^LOW^ for the remainder of this paper.

### 2.4. Cell Viability

Cell viability was evaluated using a resazurin-based assay measuring cellular reducing potential, a proxy for cell health. Wild-type (WT) or PERK^LOW^ HEK293T cells were plated on poly-lysine (Sigma-Aldrich, St. Louis, MO, USA)-treated 96-well plates at a density of 1 × 10^4^ cells per well. At 0, 24, 48, and 72 h post culture, supernatant was removed and replaced with new media containing alamarBlue reagent (AbD Serotec, Kidlington, UK) at a 1:10 dilution [45]. Cells were then incubated for 2 h at 37 °C. Absorbance was measured at 570 nm using a Molecular Devices SpectraMax Plus 384 plate reader and SoftMax Pro v6.5 software [46]. Data shown are results of two biological replicates with three technical replicates each.

### 2.5. Virus Quantification

WT or PERK^LOW^ cells were seeded on poly-lysine (Sigma-Aldrich, St. Louis, MO, USA)-treated 96-well plates at a density of 1 × 10^4^ cells per well. After 16 h, cells were infected with LGTV or POWV, multiplicity of infection (MOI) = 1. Supernatants were collected every 24 h post infection (hpi), starting at 0 hpi and ending at 72 hpi. An immunofocus assay was used to quantify infectious LGTV and POWV release as previously described [43,44]. Data shown are results of three biological replicates with at least two technical replicates.

### 2.6. Intracellular Genome Quantification

RNA was isolated from LGTV-infected cells from the previously described infection time-course experiment. After infectious supernatant was removed, cells were washed three times with Dulbecco’s phosphate buffered saline (DPBS) (Life Technologies, Carlsbad, CA, USA). RNA was then isolated using an RNeasy kit (Qiagen, Germantown, MD, USA) following the manufacturer’s instructions. Total RNA was quantified using a NanoDrop spectrophotometer (Thermo Fisher, Waltham, MA, USA), and cDNA was generated using iScript cDNA synthesis kit (BioRad, Hercules, CA, USA), using random hexamers for (+) strand genome generation and (-) strand specific primers for (-) strand genome generation. Total genome copies were quantified by qPCR using a LGTV plasmid stock of known concentration as previously described [47]. Data shown are results of three biological replicates with three technical replicates each.

### 2.7. Protein Analysis

HEK293T cells were seeded to a density of 8 × 10^5^ cells per well in six-well plates. After 16 h, cells were mock infected with complete media or infected with LGTV at an MOI of 10. For time-course studies, whole cell lysates were collected at 24, 48, and 72 hpi. WT cells treated with 10 µg/mL Tunicamycin (Sigma T7765, St. Louis, CA, USA) for 21 h prior to lysate harvest were used as a positive control for UPR activation assays. Cells were pelleted in DPBS and lysed using 300 μL radioimmunoprecipitation assay (RIPA) buffer (25 mM Tris, 150 mM NaCl, 0.1% SDS, 0.5% sodium deoxycholate, 1% Triton X-100), and a cOmplete mini protease inhibitor cocktail (Roche, Basel, CH). Aliquots were saved for protein quantification via a Pierce BCA assay (Thermo Fisher Scientific, Waltham, MA, USA). Cold methanol (−20 °C) was added to samples at a ratio of 4:1. Following an incubation no shorter than 24 h at −80 °C, precipitated samples were spun at 16.2 × 10^3^ relative centrifugal force (rcf) for 30 min to pellet protein. Methanol was then removed, and samples were reconstituted to 5 µg/µL concentration in 2× protein sample buffer (125 mM Tris, 6 mM EDTA, 10% SDS, 10% Glycerol, 0.04% bromophenol blue, 100 mM DTT) and incubated for 10 min at 95 °C.

For preparation of samples to examine autophagy by western blot, supernatants were removed 24 hpi and replaced with either fresh complete media, Earle’s balanced salt solution (EBSS) media (Sigma Aldrich, St. Louis, MO, USA), DMEM plus 10 µM bafilomycin A1 (BafA1) (Sigma Aldrich, St. Louis, MO, USA), or EBSS plus 10 µM BafA1. After 4 h, the medium was removed from cultures and whole cell lysates were made as noted in the previous section.

Proteins were separated by SDS-PAGE and transferred onto polyvinylidene difluoride (PVDF) membranes by using an iBlot gel-transfer device (Thermo Fisher Scientific, Waltham, MA, USA). Membranes were blocked with 5% nonfat milk in Tris-buffered saline–0.5% Tween 20 (TBS-T) and then incubated with primary antibodies overnight at 4 °C. After three washes with TBS-T, the membranes were incubated with secondary horseradish peroxidase-conjugated antibodies, developed with ECL Plus Western chemiluminescent system (Thermo Fisher Scientific, Waltham, MA, USA) and exposed to film. Between 2 and 4 biological replicates of each blot were performed.

### 2.8. Immunofluorescence (IF) and Image Analysis

HEK293T cells were seeded to a density of 4 × 10^4^ cells per well in eight-well Lab-Tek chambered glass slides pretreated with poly-D-lysine. After 16 h, cells were mock infected with complete media or infected with LGTV at an MOI of 10. Following 24 h, supernatant was replaced with fresh complete media, EBSS media, complete media plus 10 µM BafA1, or EBSS plus 10 µM BafA1. After 4 h, the medium was removed from cultures and examined by IF.

Cells were fixed using cold 100% methanol for 15 min, quenched with 0.1% glycine in 1× PBS, permeabilized with 0.1% Triton-X 100 for 5 min, and blocked with 1% bovine serum albumin (BSA) in 1× PBS for 1 h. Primary antibodies against LC3 and LGTV nonstructural protein 3 were added overnight at 4 °C in 1× PBS plus 1% BSA. Secondary antibodies were added for 1 h in 1× PBS plus 1% BSA and cells were mounted in Prolong Gold antifade plus DAPI (Thermo Fisher, Waltham, MA, USA). Slides were imaged using a Zeiss LSM 710 confocal laser scanning microscope. Autophagosome analysis was performed in ImageJ. Briefly, cells per field of view were enumerated by DAPI segmentation and probable autophagosomes were called using LC3 signal using size and pixel-intensity parameters. Images are a result of one biological replicate in which more than 1500 individual cells were analyzed.

## 3. Results

### 3.1. LGTV Infection Activates the UPR

**Figure 1:**
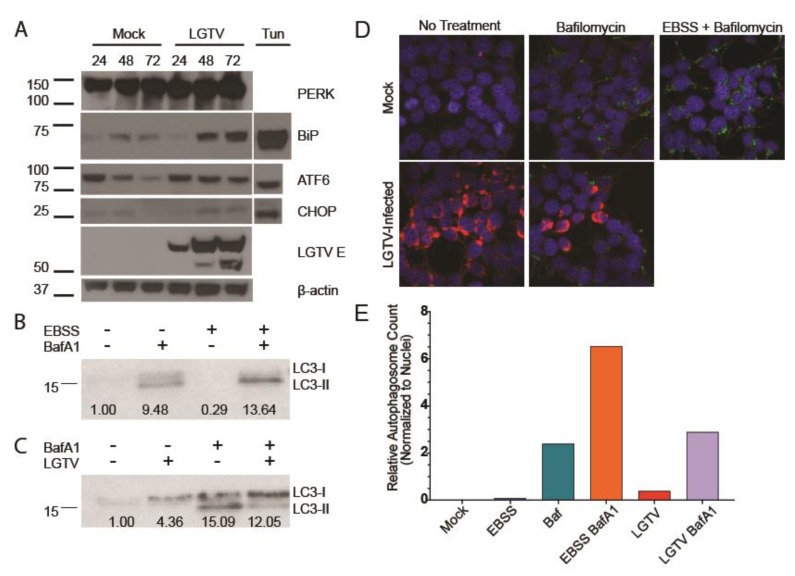
The tick-borne flavivirus Langat virus (LGTV) infection activates host unfolded protein response (UPR) and autophagy pathways. (**A**) LGTV infection (MOI = 10) in HEK293T cells generated an increase in binding immunoglobulin protein (BiP) and CCAAT-enhancer-binding protein homologous protein (CHOP) expression, but not activating transcription factor 6 (ATF6) cleavage, suggesting active protein kinase R-like endoplasmic reticulum kinase PERK signaling. Tunicamycin (10 µg/mL) control included for BiP, ATF6, and CHOP. LGTV E expression increased throughout infection time course. (**B**) Basal autophagic flux of HEK293T cells measured by microtubule-associated protein light chain 3 (LC3)-I and LC3-II expression. Earle’s balanced salt solution (EBSS) starvation increases flux, depleting LC3-I and LC3-II. Blocking autolysosomal degradation with bafilomycin A1 (BafA1) increased the abundance of LC3-II, more so under EBSS starvation. Total signal normalized to mock control and reported below bands. (**C**) LGTV infection (MOI = 10) alters autophagic flux in HEK293T cells 24 hpi (hours post infection). Infected samples had higher overall LC3 expression, but the majority was unlipidated LC3-I, suggesting inhibition of LC3 lipidation. (**D**) Representative images of HEK293T cells treated with EBSS, BafA1, or LGTV infection. DAPI represented in blue, LC3 in green, and LGTV NS3 in red. Images acquired at 63x magnification. (**E**) Average autophagosomes per cell identified by LC3 fluorescent signal normalized to number of nuclei. Very few autophagosomes recorded in non-BafA1-treated samples.

The UPR has been postulated to play a role in infections of several vector-borne flaviviruses, but no experiments to date have investigated a role in infections by LGTV. Therefore, we first examined whether LGTV activates the UPR. HEK293T cells were infected with LGTV, MOI = 10, and whole cell protein lysates were collected at 24, 48, and 72 hpi. BiP expression, a broad indicator of UPR activity [48], was measured by western blot. BiP levels increased notably throughout the infection time course (Figure 1A). This result demonstrated that LGTV infection robustly activated the UPR pathway. A slight increase in BiP expression was also observed in the mock sample at later time points.

To determine whether PERK was activated by LGTV infection, we measured levels of CHOP, a transcription factor able to bypass eIF2α-induced translation shutoff. (Figure 1A). CHOP can lead to deleterious outcomes including apoptosis [49] and we have previously described that LGTV infection induces a cytopathic effect in HEK293T cells [47]. Levels of CHOP increased as infection progressed compared to mock samples, while levels of PERK remained consistent (Figure 1A). ATF6 activation has also been reported to induce CHOP expression, so we measured ATF6 cleavage. We observed ATF6 cleavage in HEK293T cells following 21 h tunicamycin treatment, but not at any timepoint during LGTV infection. These data suggested that LGTV activated PERK, which subsequently induced production of CHOP. Thus, we have demonstrated that LGTV infection robustly and consistently activated the PERK arm of the UPR.

### 3.2. LGTV Infection Alters Autophagic Flux

Published studies have also linked PERK activation to autophagic regulation [50,51,52]. Autophagy has varied and apparently contradictory effects on flavivirus replication [35]. To evaluate the role of autophagy in LGTV infection, we chose to measure the level of LC3, a marker of autophagic flux. Under standard conditions, LC3 is present throughout the cytosol in a non-active unlipidated form, LC3-I. During autophagy, LC3-I is lipidated to form LC3-II, which has an active role in autophagosome formation. By measuring total LC3 abundance and then comparing relative quantities of unlipidated LC3-I and LC3-II, one can estimate the overall autophagic flux of a cell population.

HEK293T cells were infected at an MOI of 10. After 24 h of infection, they were treated with complete DMEM or EBSS containing BafA1 or vehicle (DMSO) for 4 h. The EBSS media starves the cells and activates autophagy. This depletes both forms of LC3 as LC3-I is lipidated to LC3-II and LC3-II is degraded as the autophagosomes fuse with the lysosomes. BafA1 treatment inhibits autophagosome fusion, preventing degradation of the lipidated LC3-II. When EBSS starvation and BafA1 treatment are combined, LC3-I is still converted to LC3-II, but LC3-II is not degraded, resulting in a robust accumulation of LC3-II only. Whole-cell lysates were probed for LC3 to confirm these phenotypes with HEK293T cells (Figure 1B). We next examined LC3 expression in LGTV-infected cells. LGTV infection produced higher levels of LC3 as determined by band intensity. Yet, when we blocked autophagosome degradation, only a small accumulation of LC3-II was observed with the majority remaining in the unlipidated inactive LC3-I form, suggesting that LGTV is capable of antagonizing LC3 lipidation (Figure 1C).

We examined autophagic activity by IF. During active autophagy, lipidated LC3-II accumulates in autophagosome membranes, corresponding to puncta in cell images. Cells were subjected to the same conditions as previously including EBSS starvation, LGTV infection, and BafA1 treatment, and then stained for LC3 (green) and LGTV NS3 (red). Autophagosomes were sparse in mock and virus-infected samples, with large accumulations only present in BafA1-treated samples. (Figure 1D,E). Our combined immunoblot and IF data suggest that the virus limits lipidation of LC3-I leading to a decrease in autophagosome formation and reduced autophagic flux.

### 3.3. PERK Has an Antiviral Effect on LGTV Replication

**Figure 2:**
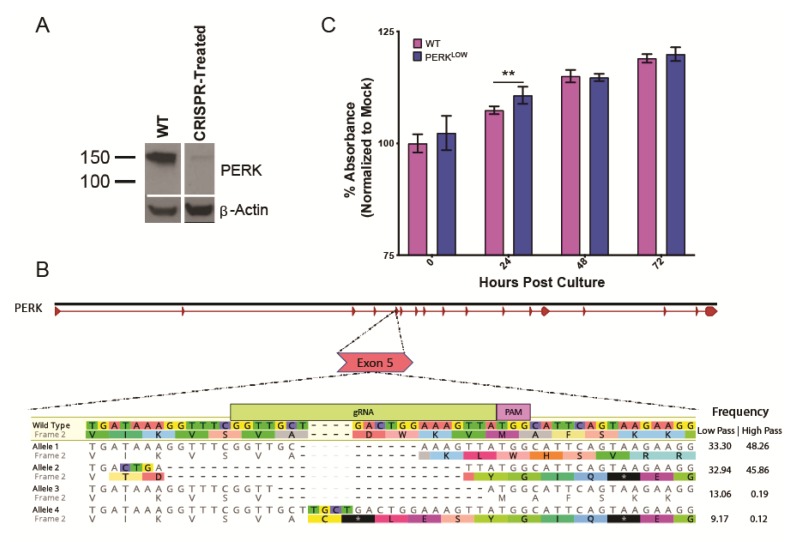
CRISPR-mediated knockdown HEK293T cell line generation. (**A**) HEK293T subclones generated from PERK-specific sgRNA express a gene product at significantly lower abundances. (**B**) Expected editing sites in exon 5 of PERK with sequencing using MiSeq. Four significant alleles were identified, three of which led to early translation termination. All edits occurred as expected in relation to the 20 nt gRNA and PAM-recognition site, denoted in green and purple, respectively. Allelic frequencies are given for both low (p. 13) and high passage (p.108) variants. (**C**) Overall reducing potential of wild-type (WT) and PERK^LOW^ cells was determined over a 72-h time course using an alamarBlue-based protocol. A slight but significant difference (~3%) was observed at 24 h. Statistical significance determined using multiple *t*-tests and a Holm–Sidak correction. ** *p*-value < 0.01

To further explore the role of PERK in TBFV infection, we knocked down PERK expression in HEK293 cells using CRISPR-Cas9 technology. We transfected HEK293T cells with a plasmid containing the Cas9 gene and an sgRNA pair targeting the fifth exon of PERK. Following 48 h, the bulk population was subcloned and individual clones were tested for PERK expression by western blot. While no clone exhibited a complete knockout phenotype, we selected one that had drastically reduced PERK expression (Figure 2A). MiSeq sequencing of exon 5 revealed four major edits corresponding to the predicted edit site as determined by the 20 nt gRNA and protospacer adjacent motif (PAM) sequence (Figure 2B). Alleles 1 and 2 represented the majority of reads and both contained deletions that led to premature stop codons. Allele 3 had a 15 nt deletion that did not shift the reading frame and allele 4 had a 4 nt insert also resulting in premature translation termination. Interestingly, sequencing of the same cell line at a higher passage number revealed loss of alleles 3 and 4. With these deletions characterized, the cell line was deemed acceptable for future experiments and for the remainder of the study will be referred to as PERK^LOW^.

We assessed whether these PERK^LOW^ cells had reduced viability by using a resazurin-based assay that measures reducing potential of cells in culture, a proxy for overall cell health. Measurements were made every 24 h for 72 h. PERK^LOW^ cells did have a slight increase in viability at 24 h, but the effect was lost at later time points (Figure 2C). Although this effect was significant, it corresponded to a miniscule increase, therefore we did not assign importance to the effect.

**Figure 3:**
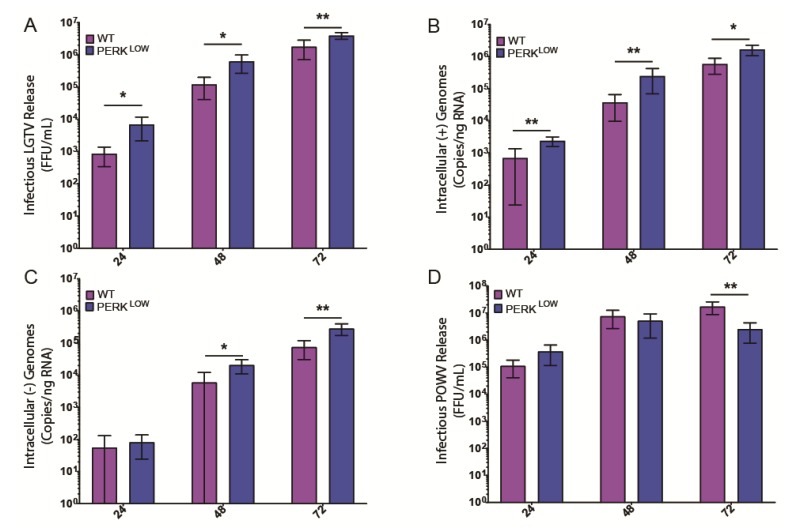
PERK has an antiviral effect on infectious LGTV release and genome replication. (**A**) PERK^LOW^ HEK293T cells released higher infectious titers at all examined timepoints compared to WT HEK293T cells following infection at MOI = 1. (**B**) PERK^LOW^ HEK293T cells produced higher quantities of (+) sense genomic LGTV RNA at all examined time points compared to WT HEK293T cells following LGTV infection at MOI = 1. (**C**) PERK^LOW^ HEK 293T cells contained higher quantities of (-) sense genomic LGTV RNA at 48 and 72 hpi compared to WT HEK293T cells following LGTV infection at MOI = 1. No difference was observed at 24 hpi. (**D**) PERK^LOW^ HEK293T cells had no significant difference in infectious virus release when compared to WT controls at 24 and 48 hpi. Lower titers were observed at 72 hpi when compared to WT HEK293T cells. Statistical significance determined using multiple t-tests and a Holm–Sidak correction. * *p*-value < 0.05; ** *p*-value < 0.01

We then used these cells to interrogate what effect, if any, PERK had on the ability of LGTV to replicate. We infected either wild-type (WT) or PERK^LOW^ HEK293T cells with LGTV at an MOI of 1. Cell supernatants and total RNA were collected at 24, 48, and 72 hpi. PERK^LOW^ cells produced eight-fold higher titers of infectious virus compared to WT and continued this trend throughout the infection time course, consistent with the MBFVs (Figure 3A). These results are consistent with the previously described antiviral role of PERK in MBFV infection (WNV, DENV, WNV_KUN_ [25,26,27]). We also measured intracellular LGTV genome abundance of (+) and (−) strands using qPCR. Copies of the positive sense, virion genome strand correlated with the infectious virus titer data, with PERK^LOW^ cells harboring higher genome copies at all examined time points (Figure 3B). Copies of the negative-sense strand, an obligate intermediate of genomic replication, showed a similar trend (Figure 3C). The combined increase in infectious virus and genome production in PERK^LOW^ cells implicate PERK as an antiviral factor in LGTV replication.

Because LGTV is a naturally attenuated TBFV, we wanted to also assess the impact of PERK reduction on replication of a more pathogenic TBFV, POWV. In contrast to LGTV, no significant effect of PERK knockdown on POWV infectious virus titers was noted until 72 h, and at that time point output was reduced as compared to the WT cells (Figure 3D). These data suggest that POWV may have acquired a strategy to antagonize PERK-mediated restriction, although more experiments are required to confirm this result. Additionally of note, production of POWV was 10–100 times higher than that of LGTV.

### 3.4. PERK-Mediated CHOP Expression Is Important to Control LGTV Infection

**Figure 4:**
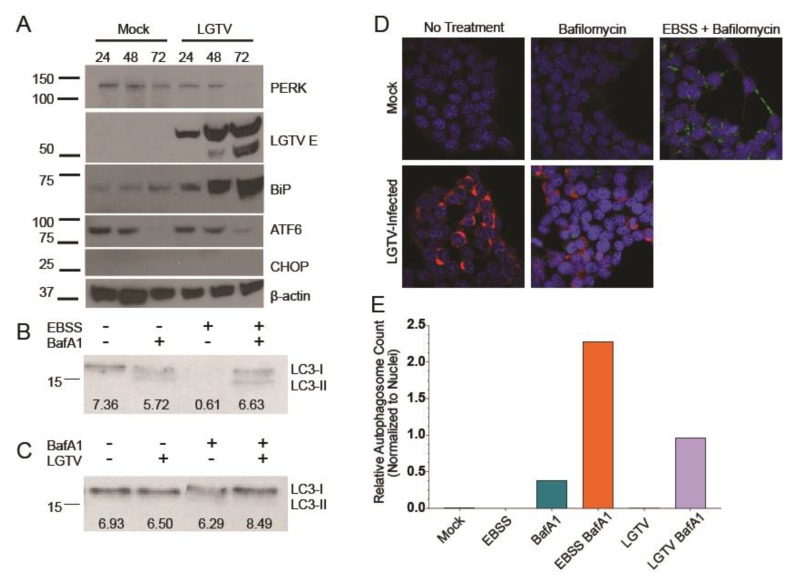
Reduced levels of PERK modify UPR and autophagy signaling during LGTV infection. (**A**) PERK^LOW^ HEK293T cells express BiP highly throughout the infection time course. No observed ATF6 cleavage or CHOP induction. LGTV E increases over infection time course. (**B**) PERK^LOW^ HEK293T cells have higher expression of LC3-I under basal conditions. Blocking autolysosomal degradation with BafA1 induced only slight increase in LC3-II showing inhibition of LC3 lipidation. Band intensity normalized to mock LC3 intensity and reported below bands. (**C**) PERK^LOW^ HEK293T have increased total LC3 expression after 24 h LGTV infection when blocking degradation although almost entirely in unlipidated LC3-I form, indicating a block in LC3 processing. (**D**) Representative images of PERK knockdown cells treated with EBSS, BafA1, or LGTV infection. DAPI represented in blue, LC3 in green, and LGTV NS3 in red. Images acquired at 63x magnification. (**E**) Average autophagosomes per cell identified by LC3 fluorescent signal normalized to number of nuclei. Fewer autophagosomes identified in PERK^LOW^ cells even following BafA1 treatment.

We next assayed the impact of reduced expression of PERK on several UPR and autophagy components in both mock- and LGTV-infected cells. Basal levels of BiP were slightly higher in the PERK^LOW^ cells, possibly reflecting a compensatory shift in UPR signaling, consequent to the reduction of PERK activity (Figure 4A). This effect has also been noted in PERK knockout mice [53]. Nevertheless, LGTV infection still dramatically induced expression, which was expected given the other sensors of the UPR were still functional. Interestingly, ATF6 activation was not increased despite the lack of PERK availability.

PERK is thought to phosphorylate eIF2α, thus attenuating translation during times of unfolded protein stress and leading to increased expression of CHOP. We consistently observed an almost complete ablation of CHOP expression in LGTV-infected PERK^LOW^ cells (Figure 4A). This finding agrees with the current MBFV literature in that PERK signaling is responsible for the bulk of CHOP expression during infection.

In addition to UPR elements, we also examined the autophagic state of the PERK^LOW^ cells by examining LC3 levels. At a basal state, more LC3-I was present than LC3-II, indicating a potential inhibition of LC3 lipidation, similar to LGTV infection (Figure 4B). When treated with BafA1, a slight increase in LC3-II was observed, but the majority of expressed LC3 remained unlipidated. This trend remained consistent with EBSS starvation conditions. LGTV infection appeared to ablate LC3 lipidation in the PERK^LOW^ cells, even with nutrient starvation (Figure 4C). We suspect this is a combinatorial effect of the lower lipidation observed under mock conditions in PERK^LOW^ cells and in LGTV infection in WT cells.

Again, we examined autophagic activity by IF, staining for LC3 (green) and LGTV NS3 (red). Greater band intensity compared to WT mock controls (Figure 1B) did not translate to higher autophagosome counts (Figure 4D,E). This is likely because the majority of signal observed in immunoblots is of the unlipidated LC3-I form, which is unable to insert into autophagosome membranes and does not aggregate into tight puncta [33]. When comparing EBSS starvation plus BafA1 treatments, the condition in which the highest number of autophagosomes are expected, we observed an approximately three-fold reduction when comparing the PERK^LOW^ cells to WT controls. Taken together, these results reaffirm both the reported role of PERK in LC3 lipidation and the ability of LGTV to inhibit this process.

## 4. Discussion

Viruses must subtly coopt host metabolism to replicate successfully while simultaneously evading the host’s innate antiviral immune responses. Several viruses, including the flaviviruses, have evolved mechanisms to suppress host immune signaling [54,55]. However, certain intrinsic stress systems, such as the UPR, can be harder to circumvent since they do not directly sense the virus. The UPR detects and responds to unusually large quantities of unfolded protein within the ER lumen, as commonly occurs during viral replication. An activated UPR response can lead to deleterious effects for both the cell and the virus, and some of the impact is mediated via the PERK arm of the UPR. Activated PERK phosphorylates eIF2α, which rapidly inhibits protein synthesis, and can lead to CHOP-mediated apoptosis. PERK also engages the autophagic system via LC3 lipidation, a key element in autophagy [50,56]. PERK signaling has been demonstrated to inhibit several mosquito-borne flaviviruses, including DENV, WNV, and WNV_KUN_ [25,26,27]. However, data regarding the role of PERK in TBFV expression is sparse.

In this study, we demonstrated that LGTV infection induces robust UPR activation, as evidenced by the dramatic increase in BiP expression (Figure 1A). PERK remained constant throughout the course of infection, but the levels of CHOP increased, indicating UPR activation through the PERK arm (Figure 1A). Finally, LGTV infection induced changes in autophagic flux (Figure 1B,C). Despite an increase in total LC3 expression upon infection, the majority remained unlipidated and was unable to aggregate in autophagosome membranes suggesting the virus somehow inhibited LC3 processing limiting overall autophagic flux.

We next showed that PERK has a clear effect on the LGTV lifecycle by infecting PERK^LOW^ cells in which we had reduced PERK expression by CRISPR technology (Figure 2A). LGTV titers reached significantly higher levels in PERK^LOW^ cells (Figure 3A). However, the same effect was not observed when examining POWV infection. In fact, by 72 hpi, PERK^LOW^ cells produced less infectious virus than WT controls (Figure 3D). A related TBFV, TBEV, also is reported to be unaffected by PERK depletion by shRNA [29]. These more pathogenic viruses may be able to better antagonize antiviral agents such as PERK. Even though no difference in growth kinetics was observed between WT and PERK^LOW^ cells at 24 hpi, both cell lines produced nearly 100-fold higher levels of infectious virus when compared to LGTV-infected counterparts. This difference in growth kinetics poses an interesting question for future inquiries and may be responsible for the more detrimental nature of POWV infection.

To determine why virus titers were higher in the PERK^LOW^ cells, we reexamined UPR and autophagy targets by western blot. BiP expression was higher in PERK^LOW^ cells, a finding we interpreted to reflect a compensatory shift in UPR signaling, consistent with previous literature. In addition, CHOP levels were much higher in infected WT cells than in PERK^LOW^ counterparts. A number of other kinases can phosphorylate eIF2α in cell stress conditions and lead to CHOP induction [57], PKR [58], heme-regulated eIF2α kinase (HRI) [59], and general control nonderepressible 2 (GCN2) [60]. We speculate that LGTV may be able to antagonize some paths of eIF2α phosphorylation, but not that of PERK. PKR is a well-characterized sensor of dsRNA, a viral replicative intermediate, and previous studies have described flaviviral inhibition or evasion of PKR-mediated eIF2α phosphorylation [61,62]. It is plausible that the direct interaction between viral dsRNA and PKR forced an evolutionary response from the virus much more robustly than the interaction between viral elements and PERK. We are currently identifying strategies to further explore this complex interplay. Differences in autophagic activity were also observed in PERK^LOW^ cells. A decrease in both LC3-II abundance by immunoblot and in autophagosome formation by IF suggest that PERK plays some role in LC3 lipidation and processing. However, similar trends in autophagic activity were seen when comparing WT and PERK^LOW^ cells infected with LGTV, suggesting decreased autophagic flux is not necessarily the cause of decreased infectious output, although further experiments are necessary to confirm.

The mechanisms explaining how different flaviviruses alter host cell programs in order to fine tune metabolism for optimal replication remain largely unknown. While this study and others increase our understanding of how these agents operate, they are restricted to single cell types and viruses. Put differently, each virus appears to possess its own mechanism, distinctive not only among related viruses, but among various cell types as well. Global techniques spanning tissues of both mammal and arthropod vectors [63] will be invaluable in unraveling the biology of these pathogens, and eventually devising ways to combat them.
